# Supplementary material for: Dual probe ligation in situ hybridization with rolling-circle amplification for high-plex spatial transcriptomics
Source: Biochem Biophys Rep. 2025 Aug 18;43:102207. doi: 10.1016/j.bbrep.2025.102207 (PMC12391569; doi:10.1016/j.bbrep.2025.102207)
Supplement: Multimedia component 1 [file mmc1.pdf]

Supplementary Figure 1

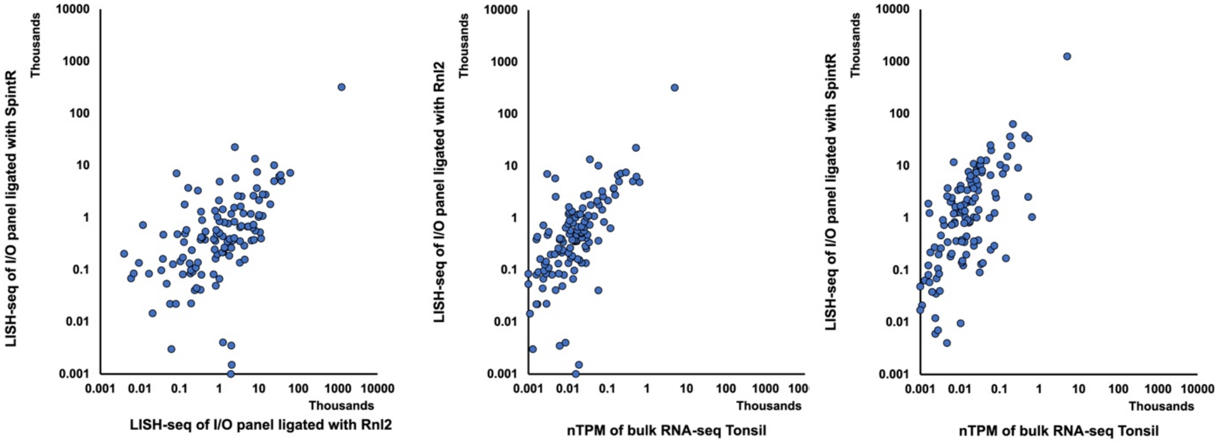

(left) Correlation between the average read count for 122 ligated probes generated from assays in which the same ligation probe sequences were ligated with SplintR (vertical axis - probes lack the diribonucleotide) or Rnl2 (x-axis - probes contain the diribonucleotide). 4 FFPE samples from tonsilitis were used for this experiment (2 samples for SplintR and 2 for RnL2). The read count for each ligated probe was averaged between the two samples. There is a significant correlation between the probesets ligated with SplintR and RnL2, r=0.99, p<0.001. While the high correlation (r=0.995) between the assays indicates that SplintR can be used as a proxy to evaluate ligation probe binding efficiencies prior to purchasing a LISH-LnR panel. Rnl2 LISH-LnR probe sets (**middle**) show slightly better correlation with bulk RNA-seq tonsil data (r=0.985, p<0.001) than fully DNA probe sets ligated by SplintR (r=0.981, p<0.001) (**right**).

Supplementary Figure 2

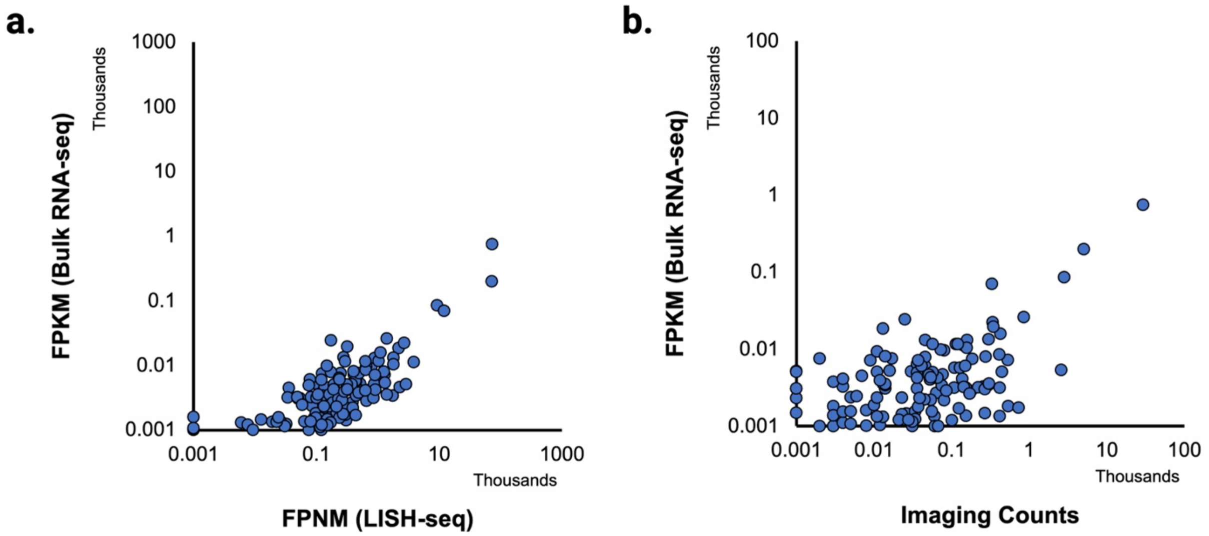

(a) Bulk RNA-seq was prepared from IBM patient samples. The LISH-LnR I/O panel was hybridized and ligated in situ*.* Ligated probes were released and sequenced. FPNM (Methods) is a modified calculation of FPKM where read counts are additionally normalized to the number of probes per gene. Bulk RNA-seq and LISH-seq data are correlated (r=0.866, p<0.001). **(b)** LISH-LnR imaging counts are correlated with bulk RNA-seq (r=0.987, p<0.001).

Supplementary Figure 3

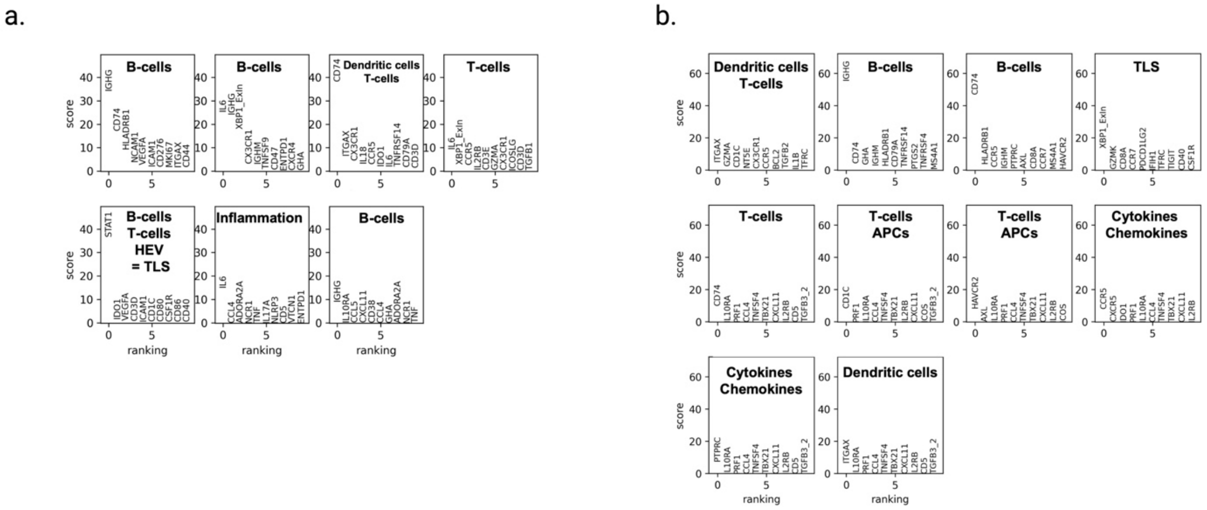

Results of the Wilcox rank tests from Leiden clustering **(a)** TLS_1 (**b**) TLS_2. Abbreviations for identified cell populations: Antigen Presenting Cells (APCs), High Endothelial Venules (HEV). Genes specific to each cluster were found using a Wilcox Rank sum test. Scores refer to ‘z-scores’ such that the higher the score, the more dominant the gene is within the cluster. 10 genes are annotated for each cluster. The cell type(s) representing each cluster were identified based on typical cellular transcriptomics signatures enhanced in oncology tissue. Both patients show clear immune activity: the presence of B-cell, T-cell types with associated inflammatory molecules, cytokines, and chemokine and chemokine receptors indicate active immune responses.

Supplementary Figure 4

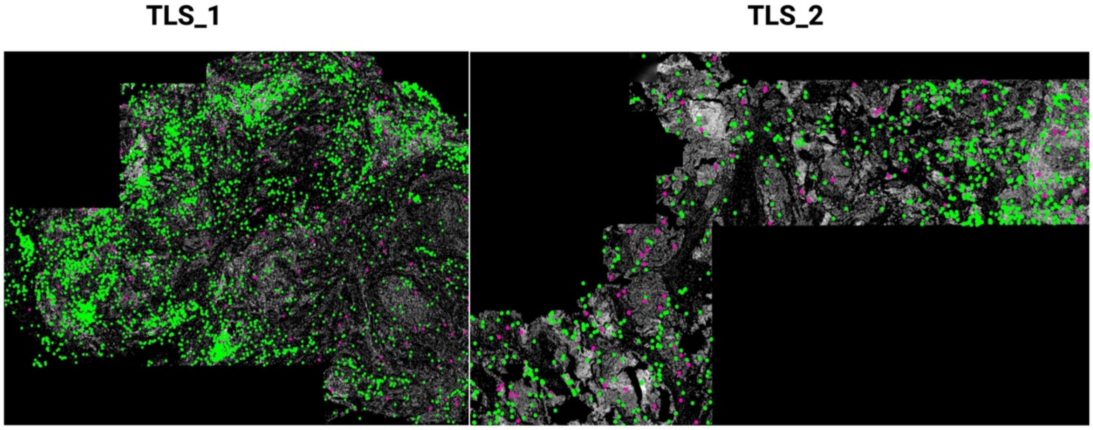

XBP1-u (green) and XBP1-s (magenta) overlays of TLS_1 (left) and TLS_2 (right). Cells in each region are stained with DAPI (shown in white).
